# Cue Integration in Categorical Tasks: Insights from Audio-Visual Speech Perception

**DOI:** 10.1371/journal.pone.0019812

**Published:** 2011-05-26

**Authors:** Vikranth Rao Bejjanki, Meghan Clayards, David C. Knill, Richard N. Aslin

**Affiliations:** Department of Brain and Cognitive Sciences, University of Rochester, Rochester, New York, United States of America; New York University, United States of America

## Abstract

Previous cue integration studies have examined continuous perceptual dimensions (e.g., size) and have shown that human cue integration is well described by a normative model in which cues are weighted in proportion to their sensory reliability, as estimated from single-cue performance. However, this normative model may not be applicable to categorical perceptual dimensions (e.g., phonemes). In tasks defined over categorical perceptual dimensions, optimal cue weights should depend not only on the sensory variance affecting the perception of each cue but also on the environmental variance inherent in each task-relevant category. Here, we present a computational and experimental investigation of cue integration in a categorical audio-visual (articulatory) speech perception task. Our results show that human performance during audio-visual phonemic labeling is qualitatively consistent with the behavior of a Bayes-optimal observer. Specifically, we show that the participants in our task are sensitive, on a trial-by-trial basis, to the sensory uncertainty associated with the auditory and visual cues, during phonemic categorization. In addition, we show that while sensory uncertainty is a significant factor in determining cue weights, it is not the only one and participants' performance is consistent with an optimal model in which environmental, within category variability also plays a role in determining cue weights. Furthermore, we show that in our task, the sensory variability affecting the visual modality during cue-combination is not well estimated from single-cue performance, but can be estimated from multi-cue performance. The findings and computational principles described here represent a principled first step towards characterizing the mechanisms underlying human cue integration in categorical tasks.

## Introduction

The problem of combining multiple sources of information (or cues) is ubiquitous: to perceive the world as a cohesive structure our brains must integrate cues within and across several modalities [1], [2]. For example, there are at least 12 different visual cues to depth [2]. A large body of prior work has shown that a speaker's facial features, such as the position of the lips or tongue, can provide useful information for the perception of spoken speech [3]–[10]. The influence of visual information on the perception of auditory information has also been observed in children [11]–[13], across languages [14]–[16] and even in pre-linguistic infants [17]–[19]. Given the extensive evidence for audio-visual integration in speech perception, the question arises as to the precise computational mechanism that is used by human observers in carrying out this integration.

**Figure 1:**
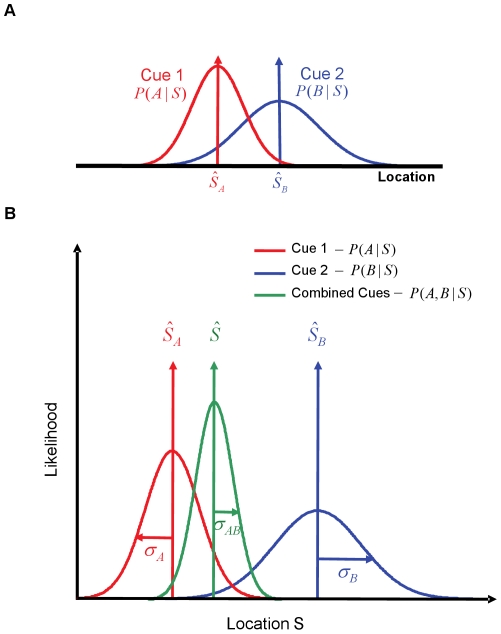
Characterizing sensory information in terms of likelihood functions. Given two sensory signals A and B, we can write the information provided by the individual signals about a stimulus S in the world, as a pair of likelihood functions, p(A|S) and p(B|S) (A) An example cue combination scenario in which the likelihood functions for the two cues are centered at different stimulus values. As a result, the cue combination task involves choosing between multiple uncertain, and conflicting, cues. (B) The likelihood function for a combination of cues is, under some independence assumptions, simply the product of the likelihood functions for each cue. This results in the peak of the joint likelihood function for the two cues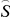, being biased toward the peak of the narrower likelihood function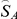. The variance of the joint likelihood function is also smaller than the variance of either of the individual likelihood function.

Under ideal conditions, where each cue is specified precisely (signals the true stimulus with perfect fidelity every time), the process of integration is trivial, because the information being signaled by each cue is exactly the same. However, in the real world, sensory signals are inherently uncertain [2], [20] and can only provide an approximation of the true stimulus. This uncertainty could be due to processing inefficiencies within each sensory modality or due to noise or variability in the environment [21]. Given such uncertainty, the information provided by a sensory cue about a stimulus in the world is best characterized by a probability distribution over possible stimulus values, the mean of which (the stimulus value that the distribution is centered on) shifts from trial to trial and across cues. This variability renders cue integration a difficult computational problem. On a given trial, any estimate drawn from the distribution representing the information provided by each cue will have uncertainty associated with it and the estimates drawn from different cues need not match each other. As a result, the brain has to infer the “true” value for the stimulus based on several uncertain sensory signals (Fig. 1A).

Contemporary research on cue integration has focused largely on the problem of estimating continuous stimulus variables from multiple sensory cues. Under certain assumptions [1], [22], [23], a statistically optimal mechanism for combining multiple uncertain cues is equivalent to using a linear combination rule where the estimate from each cue is weighted by its relative uncertainty. Formally, given two sensory signals A and B, we can write the information provided by the individual signals about a stimulus S in the world, as a pair of likelihood functions, 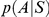
and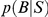. If the sensory signals are conditionally independent (e.g., the sensory uncertainty associated with each modality is independent), the information provided by both the cues together can be written as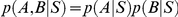. The value of S that maximizes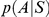 may be thought of as the estimate of S suggested by A and the value of S that maximizes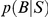 may be thought of as the estimate of S suggested by B (labeled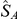 and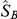 respectively). Assuming that the individual cue likelihood functions are Gaussian, the peak of the combined likelihood function can then be written as a weighted average of the peaks of the individual likelihood functions, 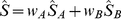
(1) where
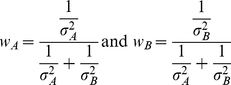
(2) and 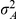 and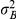 are the variances of *p(A| S)* and *p(B| S)*, respectively (when normalized over the domain of S). The variance of the combined likelihood function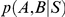 is given by:
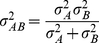
(3)


Equations (1–3) describe the information provided by a pair of cues and an estimator that computes a weighted average of the individual cue estimates (Fig. 1B). Using this weighted average comprises a normative, or statistically optimal, method for integrating the information provided by two cues for a given sensory stimulus, because the variance of the resulting estimates is guaranteed to be as small as possible (for an unbiased estimator) [20]. Numerous recent studies have shown that humans weight information from different sensory cues based on the relative sensory variability affecting them in a manner consistent with optimal integration [20], [24]–[31].

**Figure 2:**
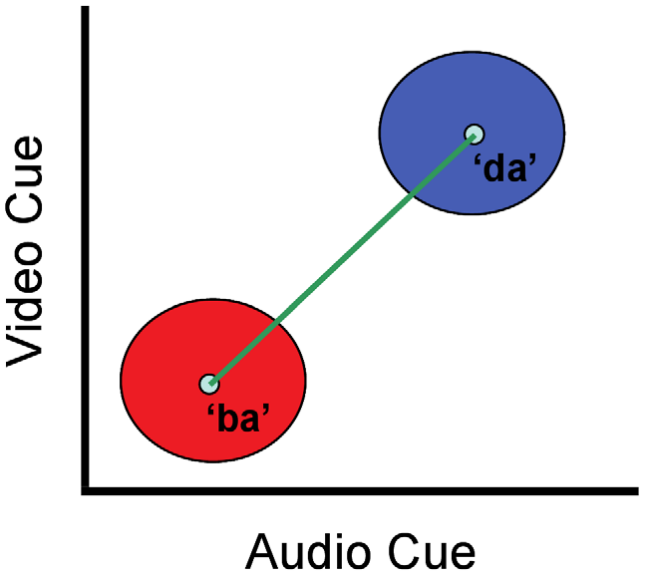
A categorical cue-combination problem. A schematic of the categorization problem where the task is to label an audio-visual speech signal as belonging to one of two phonemic categories-/ba/ or /da/. Assuming that the visual and auditory cues each vary along a single feature dimension, the x and y axes represent the strength of the sensory feature in the auditory and visual dimensions, respectively. Signals are represented as points in this two-dimensional feature space. The red and blue ellipses represent the mean and covariance of the sensory feature vectors received by an observer when a ‘ba’ or a ‘da’ is produced by a speaker in the environment, respectively. The green line represents the linear discriminant vector w that an optimal categorizer projects the received signal onto (see text).

When tasks are defined over categorical dimensions, on the other hand, they involve the added computational requirement of mapping noisy sensory features from each modality onto task-relevant categories (see Feldman & Griffiths 32] for a prior study considering the effect of categories on speech perception). Thus, the optimal cue weights in such tasks would be expected to depend not only on the sensory variability affecting each cue but also on the precise characteristics of the task-relevant categories. Consider one such categorical cue-combination problem-the task of labeling a joint audio-visual signal as a ‘ba’ or a ‘da’. For simplicity assume that the visual and auditory cues each vary along a single feature dimension. Figure 2 shows a schematic of the categorization problem. Signals are represented as points, 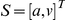
in a two-dimensional feature space, where *a* is the strength of the auditory feature and *v* is the strength of the visual feature. The red and blue ellipses represent the mean and covariance of the sensory feature vectors received by an observer when a ‘ba’ or a ‘da’ is produced by a speaker in the environment, respectively. The normative model for integrating sensory cues in such a categorization task is somewhat different from the model for integrating sensory cues when estimating a continuous stimulus value (equations 1–3 above). In a categorization task, assuming that the covariance matrices for the two categories are equivalent, the optimal categorizer 33] computes a decision variable *D* by projecting a received signal, S, onto a linear discriminant vector w (represented by the green line in Fig. 2) and labels the signal ‘ba’ when *D* is less than some criterion value *k* and ‘da’ when it is greater than *k*. If we further assume that the variance in the two sensory features is uncorrelated, the optimal mechanism for computing the decision variable D turns out to be a simple linear rule:
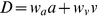
(4) where the weights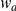 and
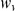
are given by 
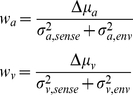
(5)

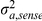
 and 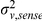 are the variances in the auditory and visual signals due to sensory uncertainty;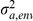 and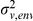 are the variances in the auditory and visual signals due to variability in environmental production (what the listeners hear), and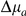 and 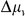
represent the separation between the means of the two categories in the auditory and visual feature dimensions, respectively. Thus, in a categorization task, the weights used to compute the optimal decision variable depend not only on the sensory variance affecting the perception of each cue but also on the precise distributional properties (parameterized by the mean and variance in the Gaussian case) of the task-relevant categories.

In order to understand the factors that determine how humans integrate auditory and visual signals for phonemic labeling, one would like to estimate all of the above parameters and compare the “optimal” weights to the weights that subjects actually assign to the two cues. Performing such a comparison, however, is a difficult problem largely because of what would be required to estimate the environmental variance (i.e. the variance in the auditory and visual signals to which a listener/viewer is exposed from speech productions in their natural linguistic environment) associated with each phonemic category. Such an estimate would require either knowing the precise distribution of all the instances of the category that the individual was previously exposed to, or being able to estimate the individual's internal model of that distribution (see Discussion).

Here, we take an alternative approach to the problem. We measure the sensory uncertainty associated with auditory and visual cues in a phonemic labeling task and use the measurements to derive a provisional normative model for phoneme categorization that computes ideal weights for each cue based only on the sensory uncertainty affecting each cue (as described in equations 1–3). If humans categorize phonemes in a Bayes' optimal way, we should see two patterns in their behavior. First, the weight that they give to a sensory cue should decrease as the sensory uncertainty in that cue increases. This is easily testable: we can experimentally manipulate the sensory uncertainty in sensory cues by degrading the stimuli presented to participants in various ways (such as by adding noise or by blurring). Second, if in addition to sensory uncertainty, participants' cue integration behavior is also influenced by the environmental variability in the sensory signals associated with each phonemic category, the change in observed weights (the weights participants assign to each cue) as sensory uncertainty is manipulated should be “flatter” than is predicted by the provisional normative model that only takes sensory uncertainty into account. This is because degrading the sensory stimulus does not change the environmental variability associated with each phonemic category. Note that our approach is somewhat different from traditional studies of cue integration. Rather than testing whether or not humans are quantitatively optimal, we test whether they behave qualitatively in the manner predicted by a Bayes' optimal observer and, finding that they do, use the Bayesian framework as an analysis tool to characterize the factors that determine participants' performance.

In this article, we describe the results of an experiment in which we measured the weights that human participants assigned to auditory and visual cues when categorizing phonemes presented along a /ba/-/da/ continuum, in an audio-visual stimulus. To examine the effect of changes in sensory uncertainty on participants' cue weights, we varied the sensory uncertainty associated with the visual cue by blurring, to varying extents, the visual signal used in the experiment. We compared the weights that participants assigned to each modality (in each blur condition) to the weights predicted by a provisional normative model that only took into account the relative sensory uncertainty associated with each cue. Participants' data confirmed both of the predictions outlined above-participants gave less weight to the visual cue as visual blur increased, but not by as much as would be predicted by the provisional normative model. Our results reveal two important findings. First, humans are sensitive, on a trial-by-trial basis, to the sensory uncertainty associated with auditory and visual cues during phonemic categorization. Second, deviations from a provisional normative model that only takes sensory uncertainty into account are consistent with the hypothesis that humans have an internal model of within-phoneme environmental variability that they factor into their categorization.

## Methods

### Ethics Statement

All experimental protocols were approved by the Research Subjects Review Board (RSRB) at the University of Rochester. Informed written consent was obtained from all participants.

### Participants

Participants were 8 monolingual native American English-speaking students from the University of Rochester. Each participant had no known hearing problems, had normal or corrected to normal vision, and was naïve to the goals of the experiment. Participants were tested individually in a quiet room over a span of four sessions-one session per day. Each session lasted approximately one hour. Participants were given the opportunity to take breaks as necessary and were paid $35 for the four sessions.

### Materials

#### Audio-only stimuli

We created a 10-step audio-only synthetic continuum between the phoneme /b/ in /ba/ and the phoneme /d/ in /da/, using the Klatt-works interface (available from Bob McMurray: bob-mcmurray@uiowa.edu) to the 1988 Klatt synthesizer [34]. We first recorded a naturally produced /ba/ and a naturally produced /da/ as spoken by a male native-speaker of American English. Endpoint stimuli were synthesized based on the parameters of these recorded syllables. All non-contrastive parameters such as pitch and syllable length were then normalized between the two endpoints. Finally, a continuum between the endpoints was created by systematically varying the onset frequency of the second formant (from 1000 to 1800 Hz) and the length of the third formant transition (from 50 ms to 100 ms) in 8 steps, which together with the two endpoints, provided a 10-step audio-only continuum. The duration of each audio-only stimulus was 367 ms (11 frames at 30 fps).

#### Video-only stimuli

**Figure 3:**
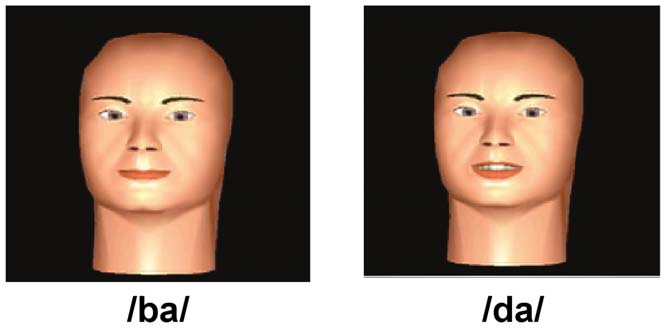
Video-only stimuli created by animating a synthetic face. Frames at voicing onset from videos of the end-point stimuli (corresponding to /ba/ and /da/) from the video-only continuum.

We also created a 10-step video-only synthetic continuum between the phoneme /b/ in /ba/ and the phoneme /d/ in /da/. This process was carried out by Dominic Massaro and his colleagues at the University of California, Santa Cruz. Massaro and his colleagues first parameterized the manner in which the position of the visible facial features (such as the position of the lips, tongue, jaw and teeth) changed during the spoken production of the syllables /ba/ and /da/. These parameters were used to animate a synthetic face to create the endpoints of the video-only continuum (Fig. 3). The parameters for the intermediate stimulus positions were obtained by linearly interpolating, in eight steps, between the parameters for the two end-point stimuli. These parameters were then used to animate the synthetic face, thereby creating the complete 10-step video-only continuum. Readers are referred to [35] for a complete description of the process by which the videos of the synthetic animated face were created. The duration of each video-only stimulus was 1.334 s (40 frames at 30 fps).

#### Audio-visual stimuli

We created 32 audio-visual stimuli by combining specific stimuli from the audio-only and video-only continua. For each audio-visual stimulus, we dubbed a chosen auditory stimulus onto a chosen video stimulus using the commercially available movie-editing software *FinalCut Pro* (documentation available online at http://documentation.apple.com/en/finalcutpro/). The duration of each audio-visual stimulus was 1.334 s (40 frames at 30 fps). The temporal alignment of the auditory and visual tracks was maintained using a dummy auditory track, included in each video file, which provided markers for the onset and completion of the auditory track. The dummy auditory track on each video file spanned 367 ms (11 frames at 30 fps) which is exactly the duration of the auditory stimuli used in this study (see above). The precise stimulus values in the auditory and visual continua that were combined to produce the 32 audio-visual stimuli are described in the results section.

#### Increasing visual uncertainty

**Figure 4:**
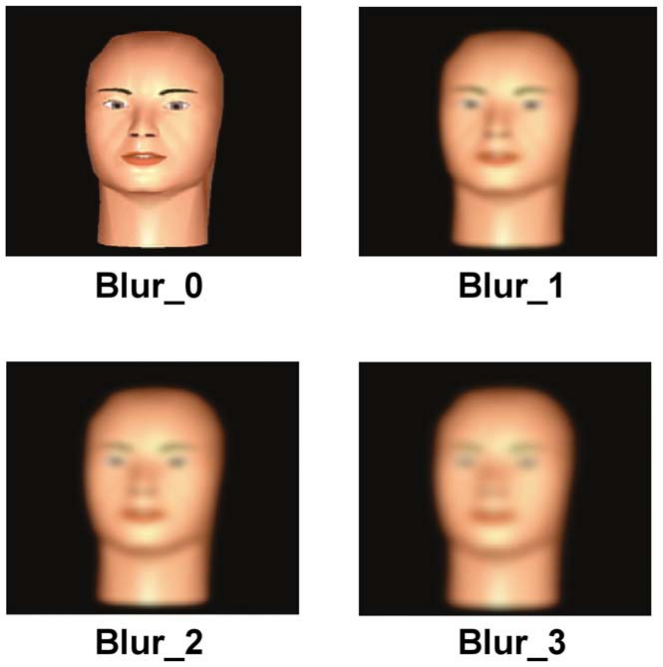
Adding blur to the visual stimuli. Frames at voicing onset from the video-only stimulus corresponding to the /da/ endpoint, showing the effect of adding increasing amounts of blur to the visual signal.

We degraded the quality of the visual information presented to participants by adding progressively greater amounts of blur to the visual stimuli, thereby creating four sets of visual stimuli (from no blur to maximum blur) (Fig. 4). Specifically, we passed each video frame of each visual stimulus through a Gaussian kernel with a specified radius, which resulted in the stimulus becoming blurred. To produce progressively greater amounts of blur, we linearly increased the radius of the Gaussian kernel, which resulted in the visual stimuli becoming blurred to a greater extent. The blurring process was carried out using the Gaussian blur routine included as part of the *FinalCut Pro* movie-editing software (see online documentation for a description of the Gaussian blur routine).

### Procedure

Participants were seated at a comfortable viewing distance from a touch-sensitive display (elo TouchSystems) that consisted of a stimulus area and two buttons-one labeled /ba/ and the other labeled /da/-and wore headphones (Sennheiser HD590). During each experimental trial, they were presented with either unimodal (audio-only or video-only) or bimodal (audio-visual) stimuli and were required to indicate whether the presented stimulus was perceived more as the phoneme /b/ in /ba/ or the phoneme /d/ in /da/, by touching the appropriately labeled button on the screen. During each session, experimental trials were divided into two blocks, where each block involved presentation of either unimodal or bimodal stimuli, with the order of stimulus block counter-balanced across sessions and participants. Each stimulus condition was repeated 26 times during the course of the experiment and, within each trial block, the specific stimulus being presented was randomly varied-within a continuum, across the two modalities and across blur levels. Each participant performed a total of 4628 trials (1157 per session) over the course of the experiment. No feedback was provided at any point in the experiment.

## Results

**Figure 5:**
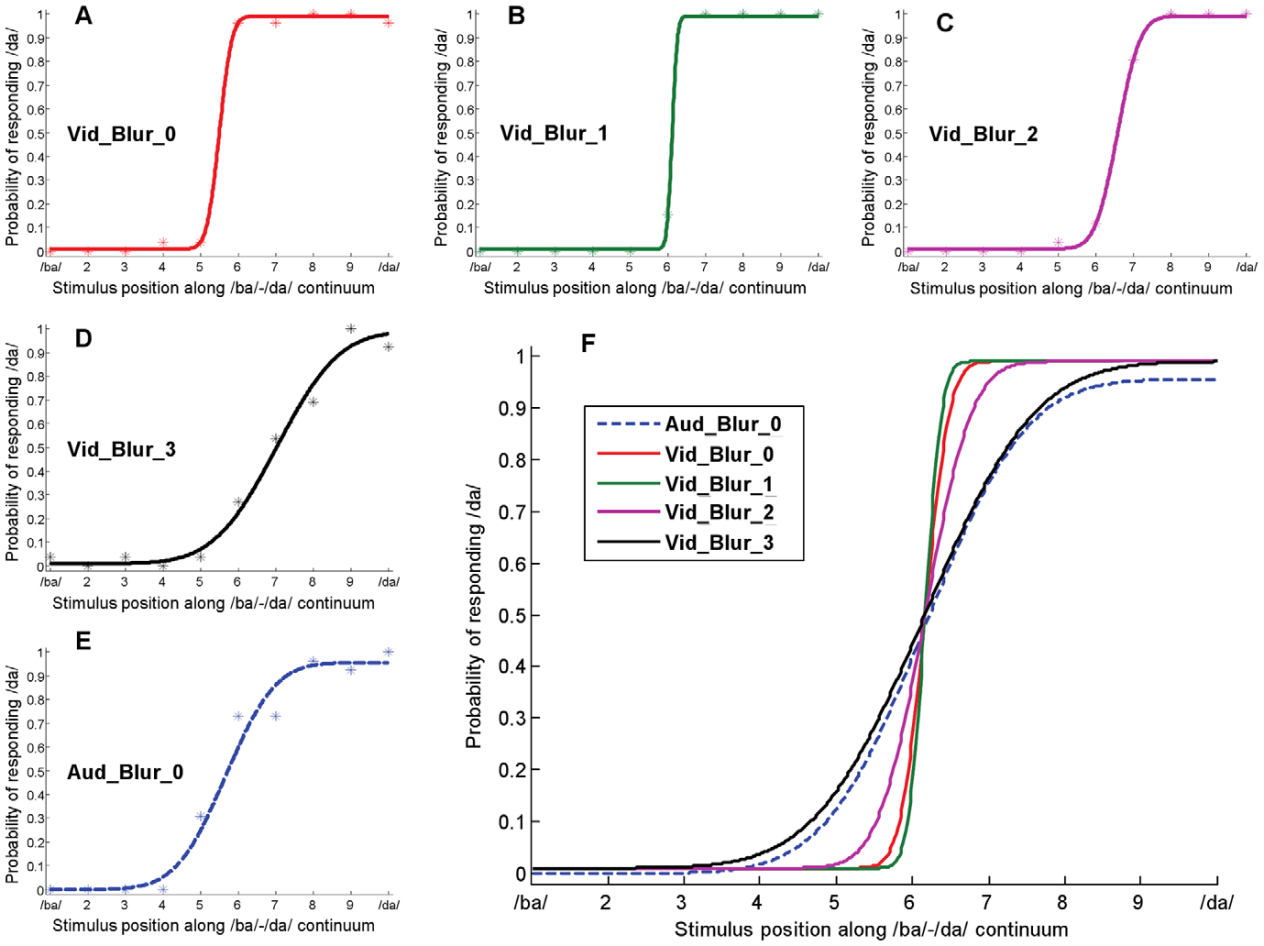
Cumulative Gaussian fits of unimodal performance, for participant ‘blh’, computed using a Maximum Likelihood procedure. The x-axis represents the unimodal stimulus continuum between the phonemes /b/ in /ba/ and /d/ in /da/. The y-axis represents the proportion of trials, for each stimulus condition, that the participant reported perceiving a /da/. The fitted functions did not need to span the entire range between 0.0 and 1.0 because we took stimulus-independent lapses into account in computing the fits. (A–E) Psychometric curves representing the participant's unimodal labeling performance were well-fit by cumulative Gaussian distributions (see text S1 for details of the fitting procedure). Panels A–D show the raw unimodal data and the corresponding fits for the four video-only conditions. Panel E shows the data and the fit for the audio-only condition. (F) Comparing the slopes of the cumulative Gaussian fits across the unimodal conditions. The point of subjective equality has been equalized in order to better illustrate the relative slopes of the psychometric functions. In the absence of any added blur in the visual signal, the slope of the video-only psychometric function (solid red line) is much steeper than that of the audio-only psychometric function (dashed blue line). However, as a greater amount of blur is added to the visual signal (green, magenta and black solid lines), the slope of the video-only psychometric function becomes shallower till at the highest blur level, the slope of the video-only psychometric function is almost the same as the slope of audio-only psychometric function.

We used a phonemic labeling task to quantitatively examine cue integration in a categorical task and to explore the extent to which human cue integration during categorical speech perception is described by the provisional normative model outlined in the introduction. To work out the predictions of the provisional normative model, we first estimated the sensory variances associated with the unimodal (audio-only and video-only) tasks, for each participant. A 10-step synthetic continuum between the phoneme /b/ in /ba/ and the phoneme /d/ in /da/ was created in both the auditory and visual domains (see Methods). Figure 3 shows the end-point stimuli (corresponding to /ba/ and /da/) in the visual domain, at the point of vocal articulation. Recall that the provisional normative model, as described in equations 1–3, predicts that for each participant in this task, the ideal weight assigned to the visual modality is a function of the sensory variability affecting visual estimates relative to the sensory variability affecting auditory estimates. By adding blur to the visual stimuli, we increased the uncertainty in the visual signal, thereby increasing the sensory variability affecting visual estimates while keeping constant the sensory variability affecting auditory estimates (Fig. 4; see Methods). Blur trials were presented randomly intermixed with blur-free trials. Psychometric curves representing each participant's unimodal labeling performance in each of the five conditions (one audio-only condition and four video-only conditions corresponding to the four levels of blur) on the 10-step continua, were well-fit by cumulative Gaussian distributions (Fig. 5; see text S1 for details of the fitting procedure). The parameters of the best fitting cumulative Gaussian for a given cue and blur condition provided the point of subject equality (PSE) and variance (slope) associated with the underlying distribution of the information provided by that cue, in that blur condition. Using the estimates for the sensory variance affecting performance in the unimodal task, we predicted the weights (via equations 1–2) that an observer, whose behavior was well-described by the provisional normative model, would assign to each modality when presented with audio-visual information simultaneously, including cue conflicts.

**Figure 6:**
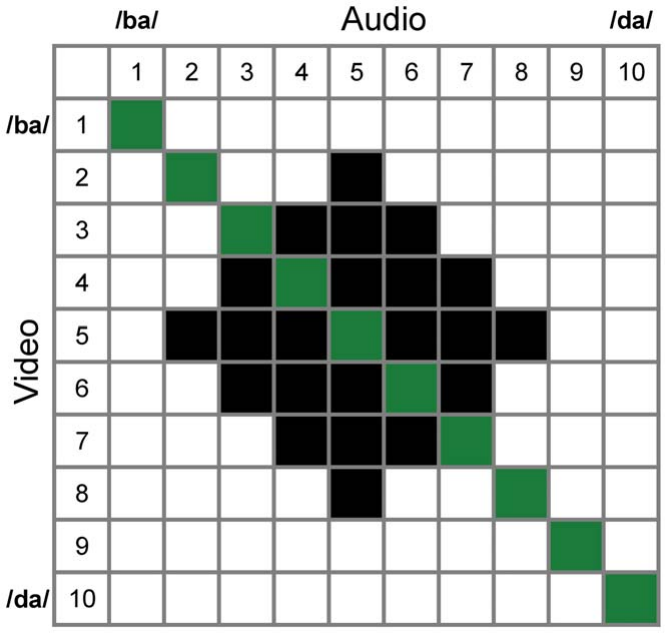
Bimodal stimuli. This figure shows the specific stimulus values from the audio-only and video-only stimulus continua that were combined to form the bimodal stimuli. Each filled square represents one of the bimodal stimuli. The stimuli highlighted in green represent the subset of bimodal stimuli that included no conflict between the two modalities. The rest of the filled squares represent bimodal stimuli that included small conflicts.

We then estimated the weights participants actually assigned to each modality during cue-combination by testing their phonemic labeling performance when presented with bimodal (audio-visual) information. 32 bimodal stimuli were created by combining stimuli from the unimodal auditory continuum with stimuli from the unimodal visual continuum (Fig. 6). Of the 32, 10 were no-conflict stimuli where the audio and video information corresponded to the same position along the unimodal 10-step continua (highlighted in green in Fig. 6; videos S1 and S2 show the cue-consistent /ba/ and /da/ bimodal stimuli), and 22 were small cue-conflict stimuli where the audio and video information was slightly offset from one another (highlighted in black in Fig. 6). To ensure that participants were not aware of these conflicts, we kept them to fewer than 3 steps (+/−3 steps) on the 10-step scale (see Discussion). We again included four different bimodal conditions, which differed in the amount of blur added to the visual stimulus (videos S3 and S4 show the cue-consistent /ba/ and /da/ bimodal stimuli, with visual blur added). The blur levels used here were the same as those used in the four unimodal conditions, and the inclusion of these bimodal conditions allowed us to compute the weight assigned to each modality in the presence of the different levels of added visual blur. Blur trials were again presented randomly intermixed with blur-free trials. Psychometric functions for each participant were fitted as in the unimodal conditions. The weights assigned by each participant to each modality were also simultaneously computed from the bimodal labeling data. Finally, we compared the weights that participants actually assigned to each modality during the bimodal task, to the weights predicted by the provisional normative model, to test the extent to which the provisional normative model describes human cue integration during phonemic labeling.

### To what extent does a normative model of cue integration, based solely on sensory variability, account for participants' cue weights during phonemic labeling?

**Figure 7:**
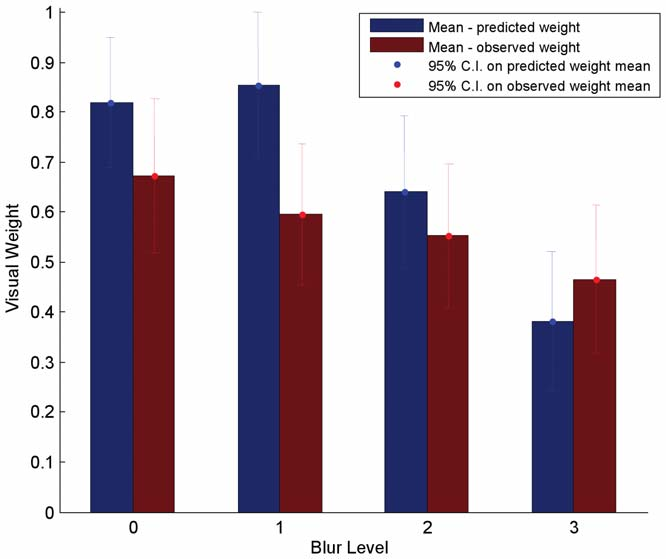
A comparison of predicted and observed weights, during audio-visual phonemic labeling. The y-axis represents weight assigned to the visual modality (or 1-weight assigned to the auditory modality). The x-axis represents the four blur levels—from no blur (Blur_0) to maximum blur (Blur_3). The blue bar, in each blur condition, represents the mean weight, across the 8 participants, that should be assigned to the visual modality if participants' behavior is well-described by the provisional normative model. The predicted weights were computed using estimates of the sensory variability affecting unimodal performance. The red bar, in each blur condition, represents the mean weight, across the 8 participants, that was actually assigned to the visual modality during the bimodal task. The error bars represent 95% confidence intervals for the respective means.

Across the 8 participants in our experiment, the provisional normative model provided an excellent qualitative description of human cue integration performance across blur levels—each cue was weighted as a function of its sensory reliability (Fig. 7). The data allowed us to make two major observations. First, in the absence of any added blur in the visual signal, the provisional normative model predicted a higher weight to the visual modality than to the auditory modality, reflecting the fact that during unimodal performance the sensory variability affecting auditory estimates was higher than the sensory variability affecting visual estimates, in our task. In line with this prediction, during cue combination, the weights assigned by participants to the visual modality were higher than the weights assigned to the auditory modality. Second, as blur was added to the visual modality, the provisional normative model predicted a decreasing weight to the visual modality (and an increasing weight to the auditory modality), reflecting the fact that the sensory variability affecting visual estimates during unimodal performance increased with an increase in added blur. In line with this prediction, during cue combination, the weights assigned by participants to the visual modality decreased significantly with increasing added blur. A repeated measures analysis of variance over the observed weights found that the weights assigned by the participants to the visual modality were significantly different across blur level [F(3,21) = 25.138, p<.0001]. Since the blur level varied randomly between stimulus presentations (i.e., blur levels were not blocked), the data suggest that participants were able to dynamically track the reliability of each modality, on a trial-by-trial basis.

### Exploring the quantitative divergence between observed and predicted weights

Although the provisional normative model provided a good qualitative description of audio-visual cue integration during phonemic labeling, it is clear from the data that the model did not provide a good quantitative description of cue integration behavior in this task-observed and predicted weights were significantly different from each other (Fig. 7). A 2-way repeated measures analysis of variance with weight type (predicted vs. observed weights) and blur level as the factors found significant main effects of weight type [F(1,7) = 18.232, p = 0.004] and blur level [F(3,21) = 45.507, p<.0001], as well as a significant interaction [F(3,21) = 17.138, p<.0001].

It is important to note that our analysis of the provisional normative model for cue integration during phonemic labeling made a critical assumption. We computed the weight that should be assigned to each cue during the cue-combination task by using estimates of sensory variability derived from performance on single-cue (or unimodal) trials. In doing so, we assumed that the variability affecting a cue's estimates during single-cue performance was the same as the variability affecting that cue's estimates during cue-combination performance. This is an assumption that has been made by most previous studies of cue integration [20], [24], [25], [27], [28], [30], [31].

However, the assumption that the same variability affects each cue's estimates during both single-cue and cue-combination performance could be violated if the perceptual judgments being carried out by the participant were different during the single-cue versus the cue-combination task. For example, the unimodal phonemic labeling task could be carried out by focusing on a subset of the underlying features in each modality. Given that the task required labeling stimuli as either /ba/ or /da/, it was possible for an observer to carry out the video-only task by just estimating the relative position of the two lips at the initial point of articulation and then mapping “closed” to /ba/ and “open” to /da/. Similarly, it was possible for an observer to carry out the audio-only task by just estimating the onset frequency of the second formant (F2) because that cue provided the acoustic basis for carrying out the /ba/-/da/ labeling task in our experiment. However, whether or not participants were able to perform the phonemic task by focusing only on a sub-phonemic feature depended on their ability to extract the relevant featural information (lip position or F2) from the signal available in each modality.

Prior evidence suggests that human observers, when presented with visual speech information and asked to make categorical judgments, are able to decompose the visual signal into its constituent features (such as lip position) and are able to use this underlying feature in isolation in carrying out perceptual tasks. On the other hand, when presented with auditory speech information and asked to make categorical judgments, human observers are unable to decompose the auditory signal into its constituent features (such as the onset frequency of individual formants) and are thus unable to use the underlying feature information in isolation [36]. Prior evidence therefore suggests that in our experiment, participants would not have the ability to extract F2 information from the auditory signal, but would have the ability to extract lip position information from the visual signal. As a result, it is possible that participants in our experiment carried out the perceptual judgment of lip-position estimation (as opposed to phonemic labeling) during the video-only task, whereas they could only have carried out the perceptual judgment of phonemic labeling during the audio-only task.

During cue-combination, since the audio-visual speech information is presented in an integrated fashion, it is unclear whether participants retained access to each cue's underlying features. If they did, then it's possible they used the same strategy in combining each modality as they did during the single-cue tasks-lip position estimation based on the visual component of the audio-visual signal and phonemic labeling based on the auditory component of the audio-visual signal. In this case, the same perceptual judgment would be carried out, in each modality, during the performance of both single-cue and cue-combination tasks and we expect the manner in which we tested the provisional normative model to be valid. On the other hand, if during the cue-combination task, participants were unable to use the individual sub-phonemic features, but instead carried out the perceptual judgment of phonemic labeling based on the entire audio-visual signal, then it is possible that different perceptual judgments were carried out in the visual modality during the single-cue versus the cue-combination task-lip position estimation during unimodal performance and phonemic labeling during bimodal performance. In such a scenario, the variability affecting visual information during the cue-combination task is not well estimated by the variability affecting visual information during the video-only task, and a key assumption made in testing the provisional normative model is violated. Thus, the quantitative divergence between the weights predicted by the provisional normative model and the weights observed from the participants (Fig. 7) need not necessarily be due to a failure of the model. Rather, it could simply be due to the fact that we used the wrong estimate of visual cue reliability in computing the predicted weights.

### Estimating variance affecting visual estimates during the cue-combination task

The discussion in the previous section leads us to the conclusion that, in our task, it is possible that participants were carrying out different perceptual judgments when presented with visual information during the unimodal versus the bimodal task. As such, in order to properly test the extent to which the provisional normative model provides a description of cue integration during phonemic labeling, we need to estimate the sensory variance affecting the information provided by the visual cue during the *actual* cue-combination task-during bimodal performance. To do this, we exploit the fact that for a given participant, the variance affecting bimodal judgments,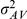, is related to the variance associated with the individual cues,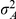 and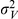. This relationship can be written as follows:
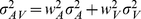
(6) where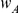 and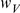 represent the weights assigned by the participant to the auditory and visual cues respectively. Equation 6 represents the relationship between bimodal variances and unimodal variances for any linear integration system, regardless of whether the cue weights are optimal. It therefore provides a means for estimating one cue's variance given reliable estimates of the other cue's variance and the bimodal variance without making any assumption of optimality. Using equation (6), we estimated the variance associated with the visual cue during bimodal phoneme categorization from the variance estimated from the cue-consistent bimodal stimuli, the weights estimated from the cue conflict bimodal stimuli, and the auditory cue variance estimated from the unimodal auditory stimuli. We compared this estimate of visual cue variance with the estimate derived from unimodal visual conditions to test the assumption, for each participant, that the sensory variance affecting visual information is the same during both unimodal and bimodal performance.

It is important to note that by using the auditory cue variance estimated from the unimodal auditory stimuli, we are assuming that the variance of the auditory estimates is the same during both unimodal and bimodal performance. As we have noted earlier, prior research [36] suggests that when presented with auditory speech information and asked to make categorical judgments, human observers are unable to decompose the auditory signal into its constituent features, thereby strongly suggesting that they are likely carrying out the same perceptual judgment of phonemic labeling during both unimodal auditory and bimodal performance. As such, if the perceptual judgment being carried out is the same during both unimodal auditory and bimodal performance, it is reasonable to assume that the sensory variance affecting auditory estimates is also the same.

**Figure 8:**
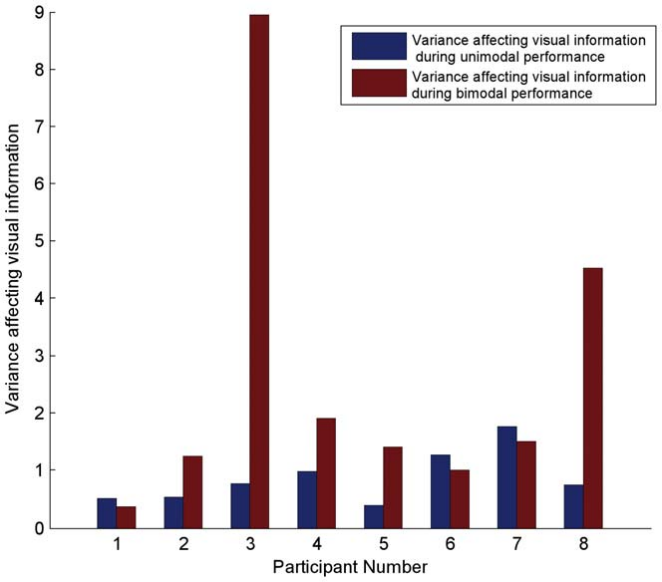
Variance affecting visual information during unimodal versus bimodal performance, for each participant. The y-axis represents the variance affecting visual information during task performance. The x-axis represents the 8 participants in our study. The blue bar, for each participant, represents the mean variance, across blur levels, affecting visual information during unimodal performance. The red bar, for each participant, represents the mean variance, across blur levels, affecting visual information during bimodal performance.

Figure 8 shows the estimates of visual cue variance derived from the unimodal and bimodal conditions, for each of the 8 participants. It is immediately apparent from this figure that for participants 3 and 8, the variance affecting visual estimates during the cue-combination task was markedly higher than the variance affecting visual estimates during the single-cue task. The reason for this dramatic difference between the two estimates of variance is unclear. Regardless of the reason, however, it is important to note that our analysis allows us to objectively examine individual participants' behavior in each component of the experiment and to tag as outliers those who grossly failed to conform to the parameters of the experiment. As a result of the above analyses, and to ensure that subsequent analyses were not unduly biased by the behavior of these outlier subjects, we excluded their data from all subsequent analyses, reducing our sample size to 6.

For the rest of our participants, it is clear that although the variance affecting visual estimates during the bimodal task is close to the variance affecting visual estimates during the unimodal task, there are nonetheless quantitative differences between the two for most of the subjects. As such, by using the variability affecting performance during the video-only task during the application of the provisional normative model, we were using the wrong source of visual variability in determining each cue's predicted weight. Thus, to properly test whether audio-visual cue integration during the phonemic labeling task is well-described by the provisional normative model, we computed predicted weights (via equations 1–2) using the variance actually affecting the information provided by each cue during the cue-combination task, as estimated according to the above analysis.

### Comparing participants' cue weights to the weights predicted by the provisional normative model

**Figure 9:**
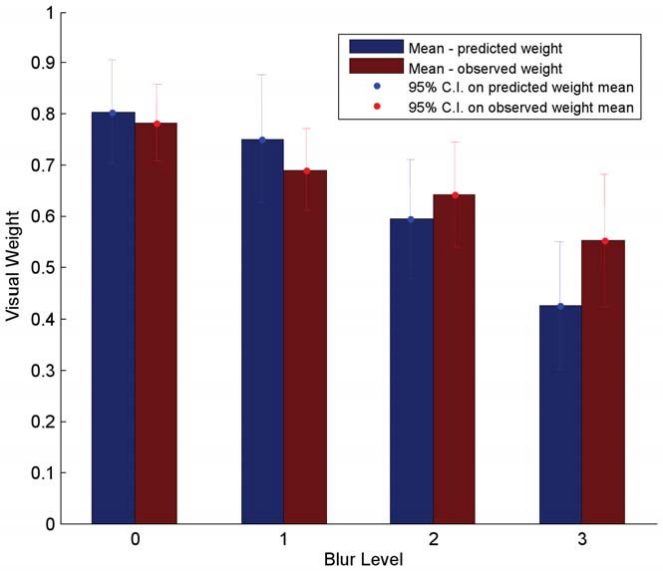
A comparison of predicted and observed weights, during audio-visual phonemic labeling. The y-axis represents the weight assigned to the visual modality (or 1-weight assigned to the auditory modality). The x-axis represents the four blur levels-from no blur (Blur_0) to maximum blur (Blur_3). The blue bar, in each blur condition, represents the mean weight, across 6 participants (excluding the two outliers from Fig. 8), that should be assigned to the visual modality if participants' behavior is well-described by the provisional normative model. This figure differs from Fig. 7 in that it shows the predicted weights, from the provisional normative model, derived by eliminating the data from the two outlier participants and by using the correct estimate of visual sensory uncertainty for each participant (see text). The red bar, in each blur condition, represents the mean weight, across the 6 participants, that was actually assigned to the visual modality during the bimodal task. The error bars represent the 95% confidence intervals for the respective means.

Figure 9 shows a comparison between the observed weights and the predicted weights, from the provisional normative model, derived by eliminating the data from the two outlier participants and by using the correct estimates of visual sensory uncertainty (i.e. the estimates of visual sensory uncertainty affecting multi-cue performance). Importantly, a 2-way repeated measures analysis of variance on the difference between the predicted weights, computed using the two prediction methods, and the observed weights confirmed that the predictive power of the provisional normative model was significantly improved by using the correct estimate of visual sensory uncertainty and by eliminating the outlier data [F(1,5) = 11.133, p = 0.021].

Across the 6 participants whose variance estimates were not grossly different between the visual-only and bimodal conditions, we found that the provisional normative model-using the correct estimate of visual sensory variability-provided a surprisingly good qualitative and quantitative prediction of participants' cue weights (Fig. 9). Specifically, in line with the predictions of the provisional normative model, we found that the weights assigned by participants to the visual modality decreased significantly with increasing blur [F(3,15) = 20.229, p<.0001]. A 2-way repeated measures analysis of variance using weight type (predicted vs. observed) as one factor and blur level as another showed no main effect between predicted and observed weights [F(1,5) = 0.324, p = 0.594]-i.e. when averaged across blur levels, predicted weights did not differ from observed weights. However, the analysis of variance revealed a significant interaction between the weight type (observed versus predicted weight) and blur-level [F(3,15) = 3.424, p = 0.045]. In particular, the effect of blur level on observed weights was smaller than predicted by the provisional normative model.

Overall, our results show that human observers integrate visual and auditory cues for phoneme categorization *qualitatively* like a Bayes-optimal observer would. The effective weight the observers give to the visual cue in our task decreases as the uncertainty in that cues increases, but that decrease is not as fast as it would be for an optimal observer who only used the relative uncertainty in the visual and auditory sensory cues to determine cue weights. Rather, this particular pattern is consistent with an observer that factors environmental variability in visual and auditory signals (as created by variability in category production within and across speakers) into its decision rule for categorizing phonemes.

## Discussion

In this paper, we explored the computational mechanisms that underlie human cue integration during categorical tasks through a quantitative analysis of audio-visual integration during phonemic labeling. Tasks in speech perception, such as phonemic labeling, are particularly interesting because they involve decisions that must be made over categorical perceptual dimensions. As we have described in the introduction, performance in such tasks should be influenced both by the sensory variability affecting each cue and by the environmental (production) variability implicit in each task-relevant category. Our data show that sensory uncertainty does indeed play a significant role in determining the relative influence of visual and auditory cues in phoneme categorization. If it were the case that the environmental variability in the two signals was the more significant determinant of performance (e.g., if the environmental variability was significantly higher than the sensory uncertainty), we would not have seen the large effect of visual blur, on the weights assigned by participants to the visual modality, that we observed in the data (see Fig. 9).

The weaker effect of blur on participants' cue weights than is predicted by the provisional normative model, which only considers our psychophysical measures of sensory uncertainty in computing the ideal weights, is what we would expect from an observer who also takes environmental variability into account (see equation 5). To illustrate the role that environmental variability might be playing in participants' cue integration behavior in our task, we fit a Bayes' optimal categorization model, that includes free parameters for environmental variance, to our participants' data (i.e. inserting our estimates of sensory variance into equation 5, we found the values for environmental variance that minimized the mean squared difference between the model weights and participants' measured weights across the four visual blur conditions). Assuming that the covariance matrices for the two categories are equivalent (a necessary assumption for the optimality of a linear categorizer-see Introduction), we find that setting the auditory environmental variance to be 18% larger than the auditory sensory variance and setting the visual environmental variance to be 42% of the level of auditory environmental variance (twice the level of the visual sensory variance in the unblurred stimulus condition) gives the best fit between the model and participants' data. This result should be interpreted with caution, as it reflects post-hoc fitting of a two-parameter model to four data points. With the caveat that the model would not be able to fit visual cue weights that change *faster* as a function of blur than is predicted by the provisional (sensory uncertainty only) normative model, it is not terribly surprising that we can find environmental variance values that provide a good fit to the human data. Nevertheless it provides at least speculative information that will be useful to future investigations that focus on the role played by environmental signal variance in audio-visual integration for phoneme categorization.

Several important contributions have been made by our findings. First, in contrast to most previous studies of human cue integration, which have only considered tasks defined over continuous perceptual dimensions (but see [37]), we have computationally and experimentally probed cue integration in a categorical task. This is an important extension of previous work because many real-world perceptual tasks involve judgments over categorical dimensions, and very little work to date has explored cue integration in such tasks. In the context of audio-visual cue integration during phonemic labeling, we specifically explored the extent to which the weights assigned by human participants to each cue depended on the relative sensory variability affecting that cue. We found that when the sensory variability affecting one of the cues was randomly varied, participants' cue weights varied in a manner qualitatively consistent with what would be predicted by an optimal categorizer. We specifically found that participants' cue weights were very similar to the weights predicted by a provisional normative model that computed ideal weights based only on the relative sensory uncertainty affecting each cue; however, they differed in an important way-they changed more slowly as a function of sensory uncertainty than would be predicted by the “sensory uncertainty only” provisional normative model. Thus, while subjects take into account relative sensory uncertainty when integrating visual and auditory cues for phoneme categorization, they are less influenced by changes in sensory uncertainty than would be expected in a world with little or no environmental variability in the signals associated with phonemic categories. This is consistent with a model in which subjects also take into account within-category environmental variability when combining cues.

Second, the techniques developed in this study allowed us to empirically explore the validity of an assumption made by most previous studies of cue integration-that the variability affecting the information provided by a cue is the same during both single-cue and cue-combination performance. This assumption allowed previous studies to use the variability affecting the information provided by each cue during a single-cue task to predict ideal cue weights during the cue-combination task. We have argued that this assumption need not be valid in all cue-combination tasks and making such an assumption is particularly problematic when considering categorical tasks, such as those in speech perception. In the context of audio-visual cue integration during phonemic labeling, we tested the validity of this assumption by independently estimating the variability affecting information provided by the visual cue during single-cue (video-only) versus cue-combination (audio-visual) task performance. Our results show that for all of our subjects the variance affecting visual information during single-cue performance was different from the variance affecting visual information during the cue-combination performance. This result provides empirical evidence that the assumption of constant variability between the single-cue and cue-combination tasks was not valid in our task. However, since our analysis implicitly provided an estimate of the variance affecting the information provided by each cue during the actual cue-combination task—during bimodal performance-we were able to properly derive the provisional normative model which thereby allowed us to quantitatively assess the role played by sensory uncertainty in participants' cue integration strategies.

Finally, we took several steps to ensure that our experimental findings are unbiased and can be generalized to other human observers. For instance, all of our experimental results were obtained by testing observers who were naïve to the purposes and motivations of the experiment. This forced us to develop analyses to empirically identify outliers and to discriminate stimulus-dependent data from stimulus-independent noise. For example, in pilot experiments we found that naïve participants had a significant stimulus-independent guessing or lapse rate, which resulted in their psychometric functions not spanning the entire range from 0.0 to 1.0. To model such stimulus-independent errors (lapses), which are known to bias the process by which participants' psychometric functions are parameterized, if not accounted for [38], we fit a modified cumulative Gaussian psychometric function to each participant's data in which the probability of selecting one of the phonemes was assumed to be a mixture of an underlying Gaussian discrimination process and a random guessing process. We also used the results of our variance estimates for each participant during the single-cue versus the cue-combination tasks to evaluate the fidelity with which participants' data conformed to the parameters of the experiment. As described earlier, this analysis allowed us to objectively examine individual participants' behavior in each component of the experiment and to tag as outliers those who grossly failed to conform to the parameters of the experiment. Finally, our experimental design generated more data per stimulus condition and participant (26 data points per stimulus condition and 4628 data points per participant) than most prior studies of speech perception. As a result, we had sufficient statistical power to make reliable inferences about individual participant performance—we fit each participant's psychometric functions separately and computed both predicted and observed weights for each participant in isolation, thereby ensuring that each participant's psychometric functions and weight estimation were unbiased by other participants' performance. This in turn allowed us to both quantitatively account for individual differences in task performance and to definitively establish our conclusions based on a relatively small sample size.

All of these foregoing steps provide a substantial advance over previous treatments of auditory-visual integration, both in terms of our experimental design and in terms of our modeling of these results. Massaro and his colleagues [5,6] have previously considered the task of phonemic categorization and have developed a model (FLMP) to describe the manner in which human participants carry out this task. However, while the FLMP captures much of the spirit of our model, our approach goes further in attempting to account for *why* listeners use the weights they do. By varying the amount of sensory variability affecting the visual modality, and then comparing participants' cue weights to the ideal weights predicted from sensory uncertainty alone, we were able to show that participants' cue integration behavior is qualitatively similar to a Bayes-optimal observer that computes cue weights based on *both* the sensory uncertainty and the environmental variability. Equally important, we only presented participants with small discrepancies between auditory and visual information (see Fig. 6), thereby avoiding the conscious awareness of qualitative mismatches between the speech signal and the visual gesture (often referred to as “fusion” in the context of the McGurk effect). A large body of prior work [39]–[41] has shown that when there are large cue conflicts in a cue integration task, participants would need to solve the “cue source” problem in addition to the cue integration problem. That is, multiple cues should only be integrated if they share a common source, and when cue conflicts are consciously apparent, participants would first need to infer whether the cues share a common source before integrating them. Experimentally, there is strong evidence that including such large cue conflicts creates non-linearities in participants' judgments about the combined stimuli, and triggers a process called “robust integration” [23,42,43]. Indeed, the classic “McGurk effect” [7] is a special case in that the cues are integrated even in the presence of very large conflicts. A model that can handle both small and large discrepancies is beyond the scope of the present report.

An important consideration in interpreting our results is that unlike traditional studies of cue integration, we did not test whether participants were quantitatively optimal in the manner in which they performed audio-visual integration during phonemic labeling. As outlined in the introduction, in order to quantitatively test “optimality”, we would need to be able to estimate the distributional properties of the phonemic categories, such as the environmental (production) variability implicit in each category and the separation in feature space between the means of the two categories, in addition to the sensory variability of each cue (equations 4–5). In order to estimate the distributional properties of the phonemic categories, we would need to either know the precise distribution of all the instances of the category that the individual was previously exposed to, or be able to estimate the individual's internal model of that distribution.

One possibility for dealing with the category separation terms (the separation in feature space between the category means -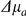 and 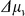
in eq. 5) would be to vary the auditory and visual signals along dimensions that are normed so that the mean separations between the auditory and visual signals are equal (thereby dropping these terms out of eq. 5). To the extent possible, we tried to do this in our study by synthesizing both the auditory and visual continua through a linear interpolation between naturally produced /ba/'s and /da/'s from native American English speakers (see Methods), and by ensuring that the points of subjective equality (PSEs) in the two continua were approximately matched. The approximately equivalent PSEs ensure that one modality was not shifted relative to the other, but we cannot be certain that the separation is precisely the same in both modalities.

It is important to note, however, that none of our conclusions depend on the separation between the category means being equivalent in the two modalities. The scale along which we measure the “/ba/-/da/ness” of a stimulus is arbitrary and is uniquely determined by the stimuli we use to define the two end points of the /ba/-/da/ continuum in each modality. Thus, the units of any measurement we make along this continuum, such as the variance of unimodal phonemic labeling performance, are relative to the scale between the two ends of the continuum. This holds true even for the estimates of the cue—weightsthe absolute estimates of these weights are determined by the scale of the continuum in each modality, regardless of the values used as the end points of the continuum-thereby allowing us to make meaningful comparisons between the sensory variance and the weights. What follows from this analysis then, is that even if the separation between the means of the two categories is different between the two modalities, as long as we use the same scale for a given modality during both the unimodal and bimodal tasks, we are guaranteed that the relative estimates of the weights will be unaffected (in particular, the comparison between the predicted weights from the provisional normative model and the weights measured from participants' actual performance). Thus, the change in weights as a function of blur and the change in weights *not* accounted for by blur, which are the focus of our interest, are entirely interpretable, given the manner in which we constructed our stimuli. Of course, as in all other experiments of this type, it is true that the absolute weights estimated in this study are only relevant for the scales that we used. That is, if we find that the weight assigned to the visual modality is greater than the weight assigned to the auditory modality, this would be true only relative to the scale used in our task, and thereby allows us to make no generalization about cue weights relative to other scales. Furthermore, if we tested a new stimulus, we could only make *absolute* predictions of how listeners would perform if we knew where that stimulus fell along the scales used in the present study.

Although our results provide clear evidence that changes in sensory uncertainty, as instantiated by blurring the visual stimuli, strongly influence the cue-weights assigned during phonemic categorization, our results also reveal an important role for the prototypical phonemic categories experienced by listeners. Estimating the environmental (production) variability implicit in each phonemic category is a particularly difficult problem because this term would be expected to depend on the precise distribution of category tokens that each participant was previously exposed to. In our task, although we did not have access to quantitative measurements of the environmental variability implicit in each phonemic category, our results are consistent with the hypothesis that participants' cue weights were influenced by environmental variability, in addition to sensory uncertainty. One way to generate quantitative estimates of the role played by environmental variability on participants' behavior in our task, as we have described earlier, is to assume that participants in our task are *quantitatively* Bayes-optimal in their cue integration behavior and to then use the Bayesian framework as a tool to calculate the influence of environmental variability on cue weights. Of course, given the limits of the data presented here, this is little more than an exercise in curve-fitting. Further work is needed to determine the general role that environmental variance plays during cue integration in categorical tasks. It might well be the case that environmental variance plays an even larger role in everyday speech recognition than is suggested in our task. For instance, although we used a two-alternative forced-choice labeling task, which is the predominant task used in almost all laboratory-based studies of speech perception, this is not the task that confronts a listener as they make judgments about words in their linguistic environment. In everyday speech recognition tasks, the number of alternative words is very large and the number of alternative phonemes is greater than two. Moreover, it is likely that production variance is much greater for some phonemes than others and in some contexts that others. It is well known that vowel productions differ considerably both within and between talkers [44] and this variability may be greater than the production variance for the consonants in the /ba/ and /da/ syllables. It is also known that changes in speaking rate and preceding phonemic context affect the production of all speech sounds. Interestingly, Schwartz [45,46] noted that there were significant differences between individual participants in the weights given to individual modalities in an audio-visual task, even when they had similar unimodal sensitivities. These individual differences were likely due to differences in the distributional properties of the task-relevant categories across individual participants.

Future work might explore the effect of the environmental variance implicit in each category on cue integration behavior, by training participants to recognize entirely novel artificial categories (see Holt & Lotto [47] for a related study). Such a training paradigm would allow the experimenter to dictate the environmental variance implicit in each category, something that is impossible to do with natural categories. Furthermore, this ability to a-priori determine the environmental variance would allow the experimenter to *quantitatively* test the extent to which human cue integration behavior conforms to the predictions of a normative model that computes ideal weights based on both sensory and environmental noise (see Knill [43] for a related study). An alternative approach would be to experimentally manipulate the amount of environmental variance implicit in each category. Although this would not change a participant's entire experience with production variability for that category, it would affect their recent experience which may also be important. Such an approach has been used in a similar phonemic labeling task [48] and was found to affect participants' categorization behavior, although cue integration was not explicitly tested in that study.

In conclusion, our results show that humans take into account changes in sensory cue uncertainty when integrating audio-visual cues to phonemic categories. In addition, by comparing participants' cue weights to the weights predicted by a provisional normative model that only considers sensory uncertainty, we show that while sensory uncertainty is a significant factor in determining cue weights, it is not the only one, and that participants' performance is consistent with an optimal model in which environmental, phonemic category variability also plays a role in determining cue weights. Although, we have not considered the question of whether humans are quantitatively optimal in the manner in which they combine audio-visual phonemic information (since doing this would require quantitative estimates of production variability), our results represent a first step towards characterizing the computational mechanisms that underlie cue integration during categorical speech perception. Finally, we have only considered a task in the domain of speech perception in the present study, but our results should also be applicable to any perceptual task that involves categorical judgments. Domains other than speech perception are replete with categories (for example, categorical judgments are commonly made in the visual domain, especially at the basic level), and the computational principles outlined in this article can be evaluated for their widespread applicability in these domains.

## Supporting Information

Video S1 **Audio-visual /ba/.** This video shows the audio-visual stimulus, corresponding to the cue-consistent /ba/ condition. It was created by combining the /ba/ endpoint from the video-only stimulus continuum with the /ba/ endpoint from the audio-only stimulus continuum (see Methods). The unblurred video-only stimuli—animations of a synthetic face—were created by members of the Perceptual Science Laboratory at the University of California, Santa Cruz (http://mambo.ucsc.edu/psl/international.html).
(AVI)

Video S2 **Audio-visual /da/.** This video shows the audio-visual stimulus, corresponding to the cue-consistent /da/ condition. It was created by combining the /da/ endpoint from the video-only stimulus continuum with the /da/ endpoint from the audio-only stimulus continuum (see Methods).
(AVI)

Video S3 **Audio-visual /ba/ with visual blur added.** This video shows the audio-visual stimulus, corresponding to the cue-consistent /ba/ condition, with blur corresponding to the ‘Blur_2’ level (see Fig. 4), added to the visual component (see Methods).
(AVI)

Video S4 **Audio-visual /da/ with visual blur added.** This video shows the audio-visual stimulus, corresponding to the cue-consistent /da/ condition, with blur corresponding to the ‘Blur_2’ level (see Fig. 4), added to the visual component (see Methods).
(AVI)

Text S1 **Data Analysis.**
(DOC)
