# Supplementary material for: Cue Integration in Categorical Tasks: Insights from Audio-Visual Speech Perception
Source: PLoS One. 2011 May 26;6(5):e19812. doi: 10.1371/journal.pone.0019812 (PMC3102664; doi:10.1371/journal.pone.0019812)
Supplement: Text S1 — Data Analysis. (DOC) [file pone.0019812.s005.doc]

**Supplementary Information**

**Data Analysis**

For each stimulus condition, the raw response data were organized into arrays specifying the number of trials (out of the 26 presentations) on which a particular participant touched the button labeled “/da/”. In pilot experiments, we found that naïve participants had a significant stimulus-independent guessing or lapse rate, which resulted in their psychometric functions not spanning the entire range from 0.0 to 1.0. To model such stimulus-independent errors (lapses), which are known to bias fits if not accounted for , we fit a modified cumulative Gaussian psychometric function to each participant’s data in which the probability of selecting one of the phonemes was assumed to be a mixture of an underlying Gaussian discrimination process and a random guessing process. We coded each participant’s decision as follows:

Y = 0; Participants touched the “/ba/” button

Y = 1; Participants touched the “/da/” button.

The psychometric model we used was as follows:

$\begin{matrix} p\left(y_{i}=1|x_{i}\right)=\gamma+\left(1-\gamma-\lambda\right)\Gamma\left(x_{i};\mu,\sigma\right) \\ p\left(y_{i}=0|x_{i}\right)=1-p\left(y_{i}=1|x_{i}\right) \end{matrix}$ **(1)**

where$y_{i}$is the participant’s response when presented with stimulus$x_{i}$on trial $i$,$\mu$ is the mean of the cumulative Gaussian which, in terms of performance in this task, represents the Point of Subjective equality (PSE) between the two alternatives, and$\sigma$is the standard deviation of the cumulative Gaussian which, in terms of performance in this task, represents the discrimination threshold. $\gamma\text{ and }\lambda$ represent lapse rate parameters, where$\gamma$ represents the base rate of performance in the absence of a signal and$\lambda$ represents the miss rate – a reflection of the rate at which the observers respond incorrectly regardless of stimulus intensity . The lapse parameters$\gamma\text{ and }\lambda$ were assumed to be constant across blur levels within a class of trials (audio-only, video-only or audio-visual) but allowed to vary across classes and were constrained to be between 0.0 and 0.25.

*Estimating parameters for unimodal performance:* Parameters for the psychometric model ($\mu\text{, }\sigma$ and the lapse parameters$\gamma\text{ and }\lambda$) for unimodal performance were computed from maximum likelihood fits to the raw unimodal performance data for each participant. The audio-only class of trials had only one blur level (no added blur), so the likelihood of a subject making a decision$Y_{i}$on audio-only trial$i$, when the presented stimulus was$x_{i}$can be written as:

$l_{i}=\left[\left(\gamma+\left(1-\gamma-\lambda\right)\Gamma\left(x_{i};\mu,\sigma\right)\right)Y_{i}\right]+\left[\left(1-\left(\gamma+\left(1-\gamma-\lambda\right)\Gamma\left(x_{i};\mu,\sigma\right)\right)\right)\left(1-Y_{i}\right)\right]$ **(2)**

The likelihood function for the entire set of audio-only data for a given subject is then given by:

$L_{aud}=\prod_{i=1}^{N} l_{i}$ where N is the total number of audio-only trials. **(3)**

The video-only class of trials had four blur levels, so the likelihood of a subject making a decision$Y_{i,j}$ on video-only trial$i$for blur level$j$, when the presented stimulus was$x_{i,j}$can be written as:

$l_{i,j}=\left[\left(\gamma+\left(1-\gamma-\lambda\right)\Gamma\left(x_{i,j};\mu_{j},\sigma_{j}\right)\right)Y_{i,j}\right]+\left[\left(1-\left(\gamma+\left(1-\gamma-\lambda\right)\Gamma\left(x_{i,j};\mu_{j},\sigma_{j}\right)\right)\right)\left(1-Y_{i,j}\right)\right]$ **(4)**

Notice that$\gamma\text{ and }\lambda$ do not change across blur-level – we are assuming that the lapse rates are constant across video-only trials. The likelihood function for the entire set of video-only trials for a given subject is then given by

$L_{vid}=\prod_{j=1}^{4} \prod_{i=1}^{N} l_{i,j}$ **(5)**

where N is the number of video-only trials for each blur level and there are four blur levels.

*Estimating parameters for bimodal performance:* During each trial of the bimodal phonetic labeling task, participants were presented with one stimulus in the auditory modality and simultaneously with another stimulus in the visual modality. Crucially, in a subset of the bimodal stimuli, there were cue conflicts between the two modalities which allowed for the estimation of the weights that participants used in combining the two cues. Since the task under consideration is a cue-combination task and since we are assuming that the system is in a linear regime, we consider the effective stimulus in this task to be a weighted combination of the two stimuli. Parameters for the psychometric model ($\mu\text{, }\sigma$ and the lapse parameters$\gamma\text{ and }\lambda$) and the weights assigned to each modality ($w_{v}\text{ and }w_{a}$) for bimodal performance were computed from maximum likelihood fits to the raw bimodal performance data for each participant. Specifically, the bimodal class of trials had four blur levels, so the likelihood of a subject making a decision$Y_{i,j}$ on bimodal trial $i$ for blur level$j$, where the presented stimulus was$x_{a_{i,j}}$in the auditory domain and $x_{v_{i,j}}$in the visual domain, can be written as:

$\begin{matrix} l_{i,j}=\left[\left(\gamma+(1-\gamma-\lambda)\Gamma\left(\left(\left(1-w_{v}\right)x_{a_{i,j}}+w_{v} x_{v_{i,j}}\right);\mu_{j},\sigma_{j}\right)\right)Y_{i,j}\right] \\ \text{ }+\left[\left(1-\left(\gamma+(1-\gamma-\lambda)\Gamma\left(\left(\left(1-w_{v}\right)x_{a_{i,j}}+w_{v} x_{v_{i,j}}\right);\mu_{j},\sigma_{j}\right)\right)\right)\left(1-Y_{i,j}\right)\right] \end{matrix}$ **(6)**

Since$w_{v}\text{ and }w_{a}$sum to one, the above expression replaces$w_{a}$ with$1-w_{v}$. Notice again that$\gamma\text{ and }\lambda$ do not change across blur-level because we are assuming that the lapse rates are constant across bimodal trials. The likelihood function for the entire set of bimodal trials for a given subject is then given by

$L_{mult}=\prod_{j=1}^{4} \prod_{i=1}^{N} l_{i,j}$ **(7)**

where N is the number of bimodal trials in each blur level and there are four blur levels.

*Avoiding local maxima when fitting psychometric functions:* Since we computed the parameters for the psychometric model for both unimodal and bimodal performance from maximum likelihood fits to the raw performance data, we needed to avoid the possibility of converging on local maxima, rather than on the desired global maximum likelihood, during the fitting process. To avoid this problem, we repeated each maximum likelihood fit five times, each time starting from a different randomly chosen initial value for the parameters. We then chose the parameters that corresponded to the fit with the best maximal likelihood value, across the five fitting runs, as the best-fit parameters for the psychometric model.

**Supplemental References**

1. Swanson WH, Birch EE (1992) Extracting thresholds from noisy psychophysical data. Perception & Psychophysics 51: 409-409.

2. Wichmann FA, Hill NJ (2001) The psychometric function: I. Fitting, sampling, and goodness of fit. Perception and Psychophysics 63: 1293–1313-1293–1313.
